# Quantification of Survival Gain From Cardiac Resynchronization Therapy: Nonlinear Growth With Time, and Greater Gain in Low-Risk Patients, Make Raw Trial Data an Underestimate of Real-World Behavior

**DOI:** 10.1016/j.jacc.2013.07.080

**Published:** 2013-12-24

**Authors:** Judith A. Finegold, Claire E. Raphael, Wayne C. Levy, Zachary Whinnett, Darrel P. Francis

**Affiliations:** ∗ International Centre for Circulatory Health, National Heart and Lung Institute, London, United Kingdom; † Division of Cardiology, University of Washington, Seattle, Washington

**Keywords:** cardiac resynchronization therapy, follow-up studies, lifespan gain, survival, CRT, cardiac resynchronization therapy, NNT, number-needed-to-treat, QALY, quality-adjusted life-year

## Abstract

**Objectives:**

The goal of this study was to examine the impact of calculation-window duration on lifespan gain (as observed in trials) and on who gains most.

**Background:**

The landmark trials of biventricular pacing (cardiac resynchronization therapy [CRT]) typically ran for <1 device battery life, and they may therefore underestimate lifespan benefit over longer durations.

**Methods:**

We conducted a meta-analysis of biventricular pacing trials to calculate lifespan gained: first, within the duration of randomized controlled trial data up to 2 years; second, over a 5-year typical battery life; and third, over >1 battery life. Importantly, we applied the Gompertz method for age-related increase in mortality from non–CRT-preventable causes.

**Results:**

Five landmark trials (COMPANION [Comparison of Medical Therapy, Pacing, and Defibrillation in Heart Failure], CARE-HF (CArdiac REsynchronization–Heart Failure), MADIT-CRT [Multicenter Automatic Defibrillator Implantation Trial With Cardiac Resynchronization Therapy], REVERSE [Resynchronization Reverses Remodeling in Systolic Left Ventricular Dysfunction], RAFT (Resynchronization–Defibrillation for Ambulatory Heart Failure)) provided data for 2 years (6,561 patients), with an average hazard ratio of 0.71. Lifespan gained across all trials increased nonlinearly with time from 0.1 month at 1 year, to 0.5 month at 2 years, and a projected 6.5 months at 5 years (65 times more than at 1 year). After multiple devices, it reached 14 months, involving on average 1.6 devices (i.e., 8.8 months per device implanted). Moreover, while over a short window (e.g., 2 years), lower-mortality patients may gain less than higher-mortality patients (1.4 vs. 2.3 months), their positions reverse by 15 years (16.0 vs. 13.7 months).

**Conclusions:**

Lifespan gain from biventricular pacing rises nonlinearly with time. Early on, higher-risk patients exhibit more gain, but later, lower-risk patients exhibit more gain. Quantifying gain over less than a patient’s lifetime underestimates lifespan gain. Over the first 1 or 2 years, lower-risk patients may seem to gain less, although they may ultimately be the ones who gain the most.

Healthcare systems need to quantify the survival benefit of interventions such as biventricular pacing (cardiac resynchronization therapy [CRT]) (1) and often discuss which patients gain most (2). Clinicians commonly use metrics such as relative risk reduction and number-needed-to-treat (NNT), although there are more sophisticated variables available such as quality-adjusted life-years (QALYs). For patients, however, the most easily understood metric is additional lifespan gained.

Lifespan gain can be evaluated from trial data. It is the area between survival curves for the device and nondevice arms. However, trials rarely run to the lifespan of the device and, even if they do, staggered enrollment means few patients with long follow-up, making the later parts of the area between survival curves noisy. Whether addressing lifespan gain over a shorter period is acceptable has not been determined for biventricular pacing. It is also unclear whether a single device should be considered, rather than a commitment to sequential devices. Competing risks from other causes of death can be expected to rise with aging, but how much does this attenuate the lifespan benefit estimated per device?

In the current study, we examined the impact of time window over which lifespan gain is quantified, on the size of that lifespan gain, and on who gains most. We conducted this analysis in 3 ways. First, within the duration of randomized controlled trials of biventricular pacing, in terms of lifespan gain per patient; second, over a typical battery life of a biventricular pacemaker; and third, over >1 battery life, quantifiable as lifespan gain per patient or per device.

## Methods

### Search strategy

We searched MEDLINE from inception to March 2013 using a combination of key words, including cardiac resynchronization therapy, biventricular pacemaker, mortality, survival, and randomized controlled trial. We also searched the bibliographies of published systematic reviews 3, 4.

Trials that compared biventricular pacing against no biventricular pacing and reported Kaplan-Meier survival curves for at least 6 months were identified. Trials with implantable cardioverter-defibrillator therapy were not excluded as long as it was present in both study arms.

### Data analysis

**Figure 1:**
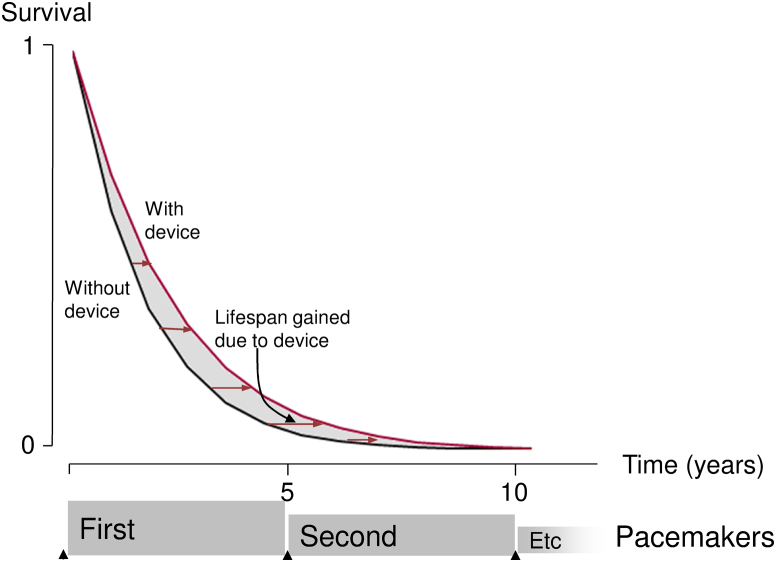
Graphic Showing How Lifespan Gained Is Related to Survival Curves Horizontal distance between the curves is lifespan gained. The distance is often greater farther down the graph (less sick patients, who survived longer). The total area between the curves is the average lifespan gained per patient.

We quantified at 3-month intervals the segmental area between the 2 curves (Fig. 1) and the cumulative area representing lifespan gained per patient up to that time.

### Effect of analysis duration on lifespan gained

The shortest duration of survival curves presented by all trials was 2 years. To examine how lifespan gained changed with follow-up duration, we calculated for each trial the lifespan gained at each time point as a proportion of lifespan gained in that trial at 2 years. The results for different trials were then scaled to permit easy comparison between trials with different mortality rates (5).

### Calculated survival gain for the duration of 1 device

Hazard ratios reported by trials are the most precise estimate of the mortality effect of the decision to implant. Making the assumption that they remain similar during the life of 1 device, we then calculated survival curves and lifespan gain to 5 years.

### Calculated survival gain for >1 sequential device

It is excessively pessimistic to halt analysis at the duration of 1 device (5) because it ignores the subsequent lifespan gain of patients who were enabled to survive until then. Conversely, during use of subsequent devices, patients are older and have more competing risks that a biventricular pacemaker may not reduce. Thus, we partitioned risk into that on which the pacemaker might have a physiological effect and that on which it would not. The pacemaker-relevant risk component was conservatively considered to remain constant with age, and the relative risk reduction for that component was also considered constant with age. In contrast, the nonpacemaker-relevant risk component was made to rise progressively with time in the standard Gompertz manner (6), and the risk reduction for that component was kept at zero.

## Results

### Characteristics of included trials

Seven trials met the criteria of comparing biventricular pacemaker implantation with no such implantation and publishing Kaplan-Meier survival curves. Two were excluded because follow-up was ≤6 months 7, 8. The 5 eligible trials 9, 10, 11, 12, 13 totaled 6,561 patients (Table 1). All provided survival curves for at least 2 years. For the COMPANION (Comparison of Medical Therapy, Pacing, and Defibrillation in Heart Failure) trial, we used only the CRT-pacemaker and no-device arms.

**Table 1:** Characteristics of Included Studies

Study, Year (Ref. #)	Trial Participants	n	Mean Follow-Up (months)	% Male	Mean Age (yrs)	Ischemic Etiology (%)	NYHA Functional Class	LVEF (%)	Mean QRS Duration (ms)	Hazard Ratio: Mortality	Hazard Ratio: Hospitalization
CRT versus medical therapy											
CARE-HF, 2005 (9)	Patients with NYHA class III or IV heart failure and with LVEF ≤35%, a left ventricular end-diastolic dimension of at least 30 mm (indexed to height), and a QRS interval >120 ms were randomly assigned to optimal medical therapy or CRT-P	813	29.4	73	67	38	III to IV	25	160	0.64 (0.48–0.85)	0.61 (0.49–0.77)
COMPANION, 2004 (11)	Patients with NYHA class III or IV ischemic or dilated cardiomyopathy and QRS duration >120 ms were randomly assigned in a 1:2:2 ratio to optimal medical therapy, CRT-P, or CRT-D	1,520	16.5 (median)	68	67	55	III to IV	22	160	0.76 (0.58–1.01)	
CRT + ICD versus ICD alone											
REVERSE, 2008 (10)	Patients with NYHA class I or II heart failure with CRT-P or CRT-D and QRS ≥120 ms and LVEF ≤40% were randomly assigned to CRT-on versus CRT-off	610	12	82	61	43	I to II	28	156	0.40	0.39
MADIT-CRT, 2009 (13)	Patients with NYHA class I or II ischemic or nonischemic cardiomyopathy, LVEF ≤30%, a QRS duration of ≥130 ms Patients were randomly assigned in a 3:2 ratio to receive CRT plus an ICD or an ICD alone	1,820	29	75	65	55	I to II	24		1.00 (0.69–1.44)	0.59 (0.47–0.74)
RAFT, 2010 (12)	Patients with NYHA class II or III heart failure with LVEF ≤30% and intrinsic QRS duration ≥120 ms or a paced QRS duration ≥200 ms were randomly assigned to either an ICD alone or an ICD plus CRT	1,798	40	83	66	67	II to III	23	158	0.75 (0.62–0.91)	0.68 (0.56–0.83)

CARE-HF = CArdiac REsynchronization–Heart Failure; COMPANION = Comparison of Medical Therapy, Pacing, and Defibrillation in Heart Failure; CRT = cardiac resynchronization therapy; CRT-D = CRT-defibrillator; CRT-P = CRT-pacemaker; ICD = implantable cardioverter-defibrillator; LVEF = left ventricular ejection fraction; MADIT-CRT = Multicenter Automatic Defibrillator Implantation Trial With Cardiac Resynchronization Therapy; NYHA = New York Heart Association; RAFT = Resynchronization–Defibrillation for Ambulatory Heart Failure; REVERSE = Resynchronization Reverses Remodeling in Systolic Left Ventricular Dysfunction.

### Lifespan gained from device implantation averaged across all trials

**Figure 2:**
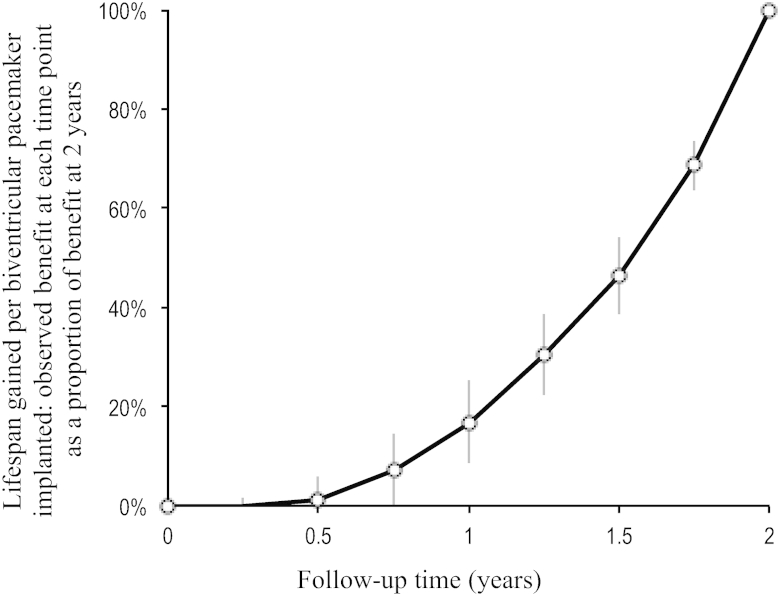
Pattern of Growth of Lifespan Gain From Device Implantation Weighted According to Study Size and Averaged Across All Trials Lifespan gained calculated at each time point as proportion of lifespan gained at 24 months. **Bars** show SEM.

The average pattern of lifespan gained, weighted according to study size between 0 and 24 months across all trials, is shown in Figure 2. Because the lifespan gain curve for each trial was rescaled to run from 0 at 0 year to 100% at 24 months, the averaged curve also does the same. The shape was nonlinear, with a slow early development and later progressively faster development of life-years gained with time. The lifespan gain at 24 months was >4 times the lifespan gain at 12 months.

### Calculated lifespan gained for the duration of 1 device

**Figure 3:**
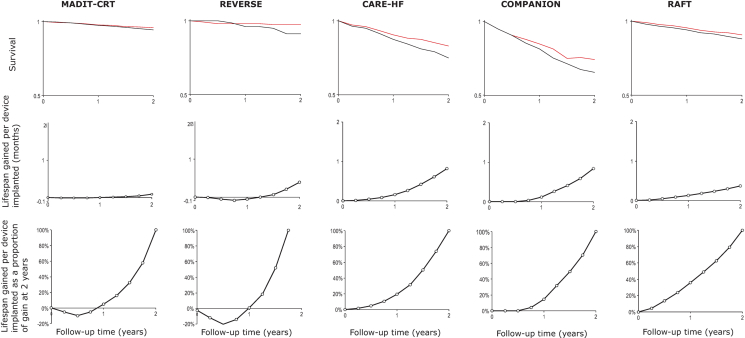
Survival, Life-Years Gained and Life-Years Gained Re-Scaled, for All 5 Trials Kaplan-Meier curves **(upper panels)**, life-years gained **(middle panels)** and life-years gained as a proportion of gain at 2 years **(lower panels)**. All 5 trials show a gradual, nonlinear increase in lifespan gain with time. CARE-HF = CArdiac REsynchronization–Heart Failure (9); COMPANION = Comparison of Medical Therapy, Pacing, and Defibrillation in Heart Failure (11); MADIT-CRT = Multicenter Automatic Defibrillator Implantation Trial With Cardiac Resynchronization Therapy (13); RAFT = Resynchronization–Defibrillation for Ambulatory Heart Failure (12); REVERSE = Resynchronization Reverses Remodeling in Systolic Left Ventricular Dysfunction.

From the trial data, Table 2 shows the lifespan gained at 1, 2, 3, and 5 years after device implantation. In parallel is shown the number of devices needed to be implanted (NNT) to gain 1 life-year. Lifespan gained rises progressively, and NNT falls progressively, for progressively longer time windows. The nonlinear pattern evident across all studies in aggregate (Fig. 2) is also visible in all 5 individual trials (Table 2, Fig. 3).

**Table 2:** Lifespan Gained and NNT to Gain 1 Life-Year Compared With Duration of Follow-Up After Device Implantation

	Lifespan Gained/Device Implanted (months)				Size of NNT to Gain 1 Life-Year			
	1 Year	2 Years	3 Years	5 Years	1 Year	2 Years	3 Years	5 Years
REVERSE	0.00	0.40			NA	30.1		
CARE-HF	0.16	0.82	2.01		75.8	14.6	6.0	
COMPANION	0.12	0.84	1.62		101.8	14.4	7.4	
MADIT-CRT	0.00	0.09	0.10		NA	137.1	119.8	
RAFT	0.13	0.37	0.77	2.52	91.3	32.7	15.5	4.8

NA = not assessable; NNT = number-needed-to-treat; other abbreviations as in Table 1.

Figure 3 illustrates the similar nonlinear pattern of lifespan gained across all 5 trials: slow early and progressively faster later. Within the 2 years available in all trials, absolute life-years gained is much larger in trials in advanced heart failure (COMPANION and CARE-HF) than in those in milder heart failure (MADIT-CRT [Multicenter Automatic Defibrillator Implantation Trial With Cardiac Resynchronization Therapy] and REVERSE [Resynchronization Reverses Remodeling in Systolic Left Ventricular Dysfunction]), as shown in the middle row of panels. However, when the curves showing development of life-years gained are re-scaled (bottom panels), a similar curvilinear progression is seen across all 5 trials.

### Calculated survival gain for >1 sequential device

In practice, patients who receive 1 device and survive its entire battery life will usually have it replaced. However, the hazard ratio with extended aging will depend on the extent to which deaths are heart failure related. If almost all mortality is heart failure related, then even substantial scaling up of the few non-heart-failure–related deaths with aging might not greatly influence the overall hazard ratio.

**Figure 4:**
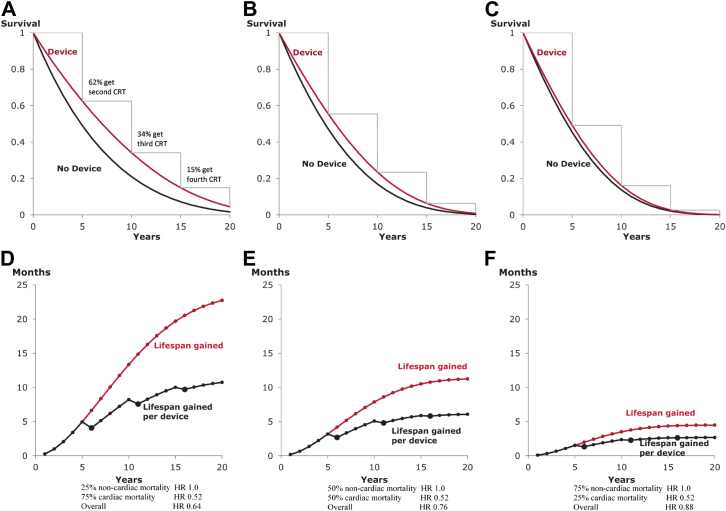
Impact of Extent of Competing Mortality Risks on Lifespan Gain From Biventricular Pacing As the proportion of non-heart-failure–related mortality is increased from one-quarter **(A, D)** to one-half **(B, E)** to three-quarters **(C, F)**, the Kaplan-Meier curves **(A to C)** become progressively closer together. Correspondingly, lifespan gain becomes progressively smaller **(D to F)**. This is true at every timepoint and regardless of whether it is calculated per patient **(red curve)** or per device **(black curve)**.

For example, in CARE-HF, one-quarter of deaths were noncardiac related. To achieve its overall 0.64 hazard ratio, the hazard ratio for cardiac death would have been ∼0.52 because (0.75 × 0.52) + (0.25 × 1) = 0.64. Had the population had a greater proportion of noncardiac deaths (e.g., 50%), then the overall hazard ratio might have been (0.50 × 0.52) + (0.50 × 1) = 0.76. Figure 4 shows the results for 3 different possibilities for hazard ratios after trial end. For Figure 4A, annual mortality is initially 12.6% (annual mortality at 1 year in CARE-HF [9]) and initially one-quarter of this is non-heart-failure related, and the absolute rate scales up by a factor of 1.1 with every year of aging, as is typical (6). For Figures 4B and 4C, initially one-half and three-quarters of mortality is non-heart-failure related and has the same age scaling.

**Figure 5:**
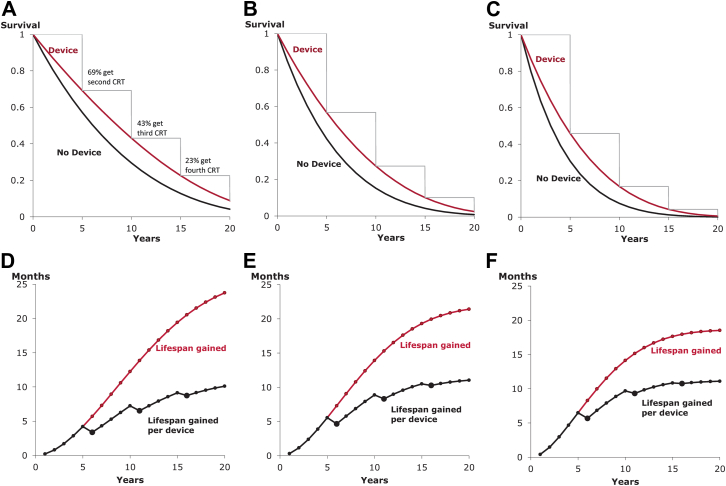
Which Risk Group Gains the Most? The answer to this question depends on the time window over which the evaluation is made. The panels show progressively increasing mortality: 10% **(A, D)**, 15% **(B, E)**, and 20% **(C, F)**; in all cases, one-quarter of the mortality is non-heart-failure related and the same initial hazard ratio from CARE-HF (0.64) is used. At the 5-year time point, the highest-risk group **(A, D)** gained the most lifespan, but at the 20-year time point, the lowest-risk group **(C, F)** gained the most.

Figure 5 shows a perhaps surprising phenomenon. For the same hazard ratio, the patient group with the highest baseline risk (Fig. 5F) exhibited more lifespan gain than the group at lower baseline risk (Fig. 5D), at the early 5-year time point. However, by 15 years, the situation has reversed. The reason for this finding is that a lower-risk group has more survivors at 5 years than a high-risk group and so continuing the calculation for longer reveals more additional lifespan gain. In our example, over a short window of 2 years, using the average hazard ratio of all 5 trials (0.71), those with a lower mortality risk (10%) would gain 1.4 months while those at higher mortality (20%) would gain much more (2.3 months). By 15 years, however, the gain in the lower-risk group increased to 16.0 months, whereas the gain in the higher-risk group reached only 13.7 months.

## Discussion

Which risk stratum of patients gains the most lifespan from biventricular pacemaker implantation depends on the time window over which gain is assessed. Over a short window, higher-risk patients may gain more lifespan than lower-risk patients, but over a longer window, this outcome is reversed. Thus, even well-designed, well-conducted, and well-reported trials of patients at lower annual risk such as MADIT-CRT (13) and REVERSE (10) ended after exposing only a tiny fraction of the full lifespan gain available from biventricular pacing.

Appropriate index of benefit depends on disease and intervention. We focused on lifespan gained, because it is relevant to patients, and on lifespan gained per device implanted because of its health/economic importance. More common metrics such as NNT and absolute risk reduction have the disadvantage of implicitly assuming no benefit after trial end; disease-modifying interventions may give continued benefit (14), which they do not capture (15). The choice of statistical metric depends on disease and intervention. For example, for brief therapies with immediate consequences (e.g., antibiotics for acute infection), NNT may be the most appropriate metric (16). For lifelong treatment with progressively accumulating cost such as drug therapy for chronic disease, a more appropriate metric is years-needed-to-treat to gain 1 life-year (15). Calculation of years-needed-to-treat must address survival gain in the posttrial period to give a correct quantification. For on–off interventions that may have sustained effect on mortality (e.g., device implantation), lifespan gain per device may be the most appropriate metric. In addition to assessing benefit in the posttrial period, this method also begins to address cost-effectiveness.

### Impact of time-window in assessing lifespan gain

Trials rarely continue with randomization intact until survival is zero in both arms; therefore, the observed lifespan gain within trials is much less than the potential gain. The only practical way of assessing lifespan gain over a satisfactorily long period is with the use of modeling, but this method must take into account the progressive increase in noncardiac mortality. We used the Gompertz method for this.

At 1 year, the lifespan gain was only (10) 0.1 month, but by 2 years this had grown to 0.5 month, by 5 years to 6.5 months, and by 15 years to 13.7 months. Such calculations are dependent on the hazard ratio being preserved in the longer term, supported by the CARE-HF (17) finding of no attenuation of hazard ratio in its extension period.

### Impact of patient group studied in assessing lifespan gain

Trials in patients at high mortality risk may have greater statistical power, but these patient groups may not be those who gain the greatest lifespan benefit over their entire lifetime, since their lifetime may be short (2). Conversely, trials of the same duration in patients at lower mortality risk are inherently less powered, but these patient groups may gain the greatest increment in lifespan if given a lifetime of biventricular pacing. Thus, even though CARE-HF and COMPANION were the trials reporting the most encouraging mortality effects from biventricular pacing, we should not assume that REVERSE and MADIT-CRT populations, if given a lifetime of pacing, would not exhibit a lifespan benefit.

When faced with patients such as those of REVERSE or MADIT-CRT, we should consider carefully whether it is wise to delay device implantation until they reach the CARE-HF/COMPANION stage of disease (18). Delaying may cause the curves to diverge quicker, but our analysis indicates it may also miss the majority of the opportunity for lifespan gain.

Although the etiology of heart failure affects lifespan gain, quantitative analysis is limited because Kaplan-Meier plots are not available for these subgroups. Gain is likely to be greater for patients with nonischemic heart failure than ischemic heart failure.

### Need for modeling to assess the cost-effectiveness of biventricular pacing

Previous analyses 19, 20, 21 exploring the cost-effectiveness of biventricular pacing have used trial data to estimate the incremental cost-effective ratios. Device implantation has front-loaded costs, and using trial data only will therefore tend to underestimate cost-effectiveness by concentrating on the early period when cost is already fully exposed but lifespan gain is only partly revealed. This pattern has also been observed for implantable defibrillators 22, 23.

### Study limitations

Our study analyzed survival to 2 years because this was the period for which all the trials showed Kaplan-Meier survival data. We used the Gompertz method to address posttrial survival although there are alternatives, including the Deale method (24). Modeling post-trial survival can only provide an estimate of the potential benefit of an intervention. However, prolonged clinical trials are expensive, and it may be ethically unacceptable to withhold devices from patients in the control arm long after the device is proven beneficial for mortality. Modeling may therefore be the best way of quantifying ultimate survival gain.

Our analysis used only total mortality data rather than attempting to assess quality of life. For formal cost-effectiveness analysis, it is usual to assess QALYs. Because patients with milder heart failure often have higher quality of life, each incremental life-year gained for them would contribute more QALYs than a life-year gained in a patient with more severe disease. Therefore, this effect of patients with milder disease showing more gain over the long horizon may be even greater when analyzed with QALY data.

## Conclusions

Lifespan gain from biventricular pacemaker implantation rises rapidly with time and much more than linearly. It continues to grow as long as patients continue to survive and are free of competing mortality risks. For this reason, although higher-risk patients may show clear gain at early time points when lower-risk patients (e.g., the MADIT-CRT [13] and REVERSE [10] cohorts) show no significant gain, this situation may reverse with time.

Such analytical approaches to quantifying lifespan gain may be useful because designing trials to directly observe lifespan gain in its entirety would need maintenance of randomization for decades.
